# Bronchoscopic Lung Volume Reduction: A Narrative Review and Proposal for the Inclusion Criteria

**DOI:** 10.3390/jcm14093190

**Published:** 2025-05-05

**Authors:** Firas Ido, Michael DiRico, Kartik Shenoy

**Affiliations:** 1 Department of Critical Care Medicine, King Faisal Specialty Hospital & Research Center, Riyadh 11211, Saudi Arabia; 2 Division of Pulmonary, Critical Care, and Sleep Medicine, University of Florida, Gainesville, FL 32611, USA; michael.dirico0516@gmail.com; 3 Department of Thoracic Medicine and Surgery, Lewis Katz School of Medicine at Temple University, Philadelphia, PA 19140, USA

**Keywords:** COPD, emphysema, bronchoscopic lung volume reduction, endobronchial valves, pulmonary function testing

## Abstract

Emphysema is an irreversible lung disease with significant morbidity and mortality, with limited treatment options in advanced stages. Recent guidelines support the use of endobronchial valves for bronchoscopic lung volume reduction in severe chronic obstructive pulmonary obstruction (COPD). Following a detailed examination of the inclusion and exclusion criteria of previously reported clinical trials, we propose expanded patient selection criteria. This proposal may increase patient referrals and patients deemed eligible for a procedure, which has been shown to decrease morbidity and mortality.

## 1. Introduction

Emphysema is an irreversible, destructive lung disease associated with significant morbidity, particularly in advanced stages [1]. Treatment options for severe disease are limited; however, recent guidelines recommend bronchoscopic lung volume reduction (BLVR) in select patients. This minimally invasive method of placing a one-way endobronchial valve has largely replaced the previous lung volume reduction surgery as the first-line intervention for patients who remain symptomatic despite maximal medical therapy. This is attributed to its decrease in morbidity and broader patient inclusions. The benefits of BLVR include improvements in symptoms (SGQR questionnaire), endurance (6 min walk distance), and pulmonary function testing (PFT). Changes in PFT include an increase in the forced expiratory volume in the first second (FEV1) and a reduction in total lung capacity (TLC) and residual volume (RV) [2]. In the subgroup of patients who undergo successful procedures and achieve lobar atelectasis, a decrease in exacerbations, a decrease in the number of respiratory failure episodes, and improved survival have also been observed [3,4,5]. A study investigating the eligibility of patients referred for BLVR found that only 19% met acceptable criteria, with 16% of those excluded lacking enough air trapping based on pulmonary function testing (PFT) [6]. Prospective studies assessing the efficacy of BLVR have differed regarding the inclusion criteria, specifically in terms of the required PFT parameters. A prerequisite to all studies was the presence of severe COPD, along with evidence of significant air trapping on PFT. It is important to note that other inclusion and exclusion criteria (intact lobar fissure with an absence of collateral ventilation, absence of lung nodules, no evidence of pulmonary hypertension, etc.) were also assessed to determine BLVR eligibility [7]. However, variations in PFT inclusion criteria may consequently lead to decreased referrals or increased patient disqualification who would otherwise benefit from BLVR. Therefore, a set of inclusion criteria for pulmonary function testing can help guide the referral process and minimize inconsistencies between centers. Here, we discuss the different PFT parameters used in several hallmark studies and review the outcomes, adverse effects, and mortality. We also review the benefits of BLVR on lung mechanics and pulmonary physiology. Based on these studies, we propose universal inclusion criteria regarding spirometry and lung volumes for BLVR.

## 2. Background

### 2.1. Pulmonary Function Test Changes in Emphysema

Understanding the benefits of BLVR requires knowledge of lung mechanics and the changes that occur in emphysema. The pathophysiology of emphysema includes chronic inflammation driven by neutrophils, macrophages, and oxidative stress. Most commonly, this can occur and be accelerated by long-term smoke exposure. The result is tissue destruction, particularly of alveolar septa and elastin fibers [8]. The loss of elastin fibers causes premature airway collapse during exhalation, resulting in a decreased FEV1 and, subsequently, air trapping, also known as dynamic hyperinflation. At rest, the destruction of elastin fibers increases airway caliber, which translates to the presence of static hyperinflation. On PFT, static hyperinflation reflects an increase in TLC, while air trapping is indicated by an increased RV. Finally, the destruction of alveolar septa impacts the alveolar capillaries, leading to a decreased diffusion capacity when assessed with inhaled carbon monoxide, termed the DLCO [9].

The hyperinflation in emphysema bears other consequences involving the respiratory muscles and chest wall. The overexpansion of the lungs at rest causes flattening of the diaphragm and, therefore, a decreased area of apposition, minimizing descent during inspiration. Hyperinflation also causes other respiratory muscles (i.e., intercostal muscles) to stretch at rest, taking on less-than-optimal positions for maximal muscle contraction. In addition, at the onset of exhalation, muscle contraction occurs when alveolar pressure is still positive due to air trapping, which increases the overall workload and energy expenditure. Finally, in heterogenous emphysema, areas of the lung most affected by air trapping can restrict neighboring lobes from fully expanding during inspiration [10].

### 2.2. BLVR Pre-Valve Assessment

Bronchoscopic lung volume reduction targets select areas of the lung most affected by emphysema. Aside from pulmonary function testing, patients must undergo additional testing to determine eligibility. Proprietary software evaluates a quantitative computed tomography (CT) of the chest to determine the degree and heterogeneity of destruction due to emphysema and fissure completeness. CT imaging is first reviewed by the practicing provider and a radiologist to conclude that emphysema is significant and there are no other contributions to pulmonary disease from processes such as bronchiectasis or fibrosis. Software-driven analysis adds an objective grade, which may be more precise and remove interobserver agreement [11]. Various densiometric parameters have been evaluated for scoring emphysema [12]. A threshold of −910 Houndsfiled units (HU) has been shown to correlate between emphysema on CT imaging and pathological emphysema in resected lungs [13]. A strong correlation has also been reported at a threshold of less than −950 HU [14].

In consideration of BLVR, the emphysema score is determined by the percentage of the lobe found to be less than −910 Hounsfield units. Consensus follows the inclusion criteria of the hallmark LIBERATE trial and considers 50% of the lung less than −910 HU sufficient to be a target lobe for BLVR, though some also consider 30% less than 950 HU as a secondary parameter [15]. Ipsilateral lobes are considered heterogeneous if there is a greater than 15% difference in emphysema score between them [16]. Fissure completeness is determined by the percentage of the length of the fissure that is visible on CT. Generally, a 95% or greater fissure completeness on imaging is considered to have a high enough likelihood that there is no collateral ventilation to proceed with the procedure. Fissure completeness of 80–94% may also be acceptable, though physiologic testing is recommended in these patients, and below 80% generally excludes patients from BLVR. An echocardiogram is performed to analyze the presence of pulmonary hypertension. A quantitative ventilation-perfusion scan is recommended to assess the degree of perfusion of each lobe to avoid targeting an area of highly increased perfusion, which would lead to shunting [15,16].

The standardized physiologic testing for fissure completeness is proprietary under the name Chartis (Pulmonx, Redwood City, CA, USA). Under bronchoscopic guidance, a balloon is inflated to occlude the desired airway, and the presence of collateral ventilation, which would prevent complete collapse, is assessed. If all criteria are met, one-way valves are deployed into a lobar or segmental bronchi.

### 2.3. Pulmonary Physiology Following BLVR

Currently, two approved endobronchial valves include the Zephyr (Pulmonx, Redwood City, CA, USA) and Spiration (Olympus, Center Valley, PA, USA) valves, which differ in design; however, both function via the same global mechanism. Once deployed within the bronchi, the valves prevent air from traveling into the target segment or lobe during inspiration but allow for expired air to exit, leading to passive deflation until complete collapse is achieved [17]. This results in decreased air trapping and hyperinflation, hence improvements in RV and TLC, respectively. There is also a larger decrease in RV compared to TLC, leading to an increase in forced vital capacity (FVC). Since FEV1 is proportional to FVC, FEV1 is expected to increase as well. Indeed, an improvement in post-bronchodilator FEV1 is the main PFT parameter typically included in the primary or secondary outcomes in studies as a measure of success. As previously discussed in the pathophysiology of emphysema, the selective collapse of an emphysematous area of the lung leads to a rise in the diaphragm along with the improved alignment of respiratory muscle fibers, which optimizes muscle contraction. These changes decrease the workload imposed on the muscles of respiration [18,19,20].

### 2.4. Comparison of Study Selection Criteria

Several prospective studies over the past decade have assessed the efficacy of BLVR (Table 1). In each study, inclusion criteria regarding pulmonary function test parameters involved specific cutoff values for the post-bronchodilator FEV1, TLC, and RV based on the percent predicted. In nearly all the prospective trials (EMPROVE, TRANSFORM, LIBERATE, IMPACT, VENT, and REACH), the inclusion criteria for post-bronchodilator FEV1 were either <45% predicted or specified as ranging between 15–45% predicted. The average pre-procedural FEV1 in these studies ranged between 27–31% predicted. Two trials encompassed slightly more liberal inclusion criteria for FEV1, which were STELVIO and BeLieVeR-HIFi, which included patients with an FEV1 of <60% and <50% predicted, respectively. In terms of lung volumes, all studies used a TLC cutoff of >100% predicted for the inclusion criteria; however, the REACH and EMPROVE trials included the value 100% predicted as well. The RV inclusion criteria had the largest variability with most studies including patients with an RV >150% predicted, except for the TRANSFORM, LIBERATE, and IMPACT trials, which used cutoff RV values of >180% predicted, ≥175% predicted, and ≥200% predicted, respectively. The diffusion capacity of carbon monoxide (DLCO) was not specified in most studies but was used to exclude patients in the VENT and LIBERATE trials if the value was <20% predicted [15,16,21,22,23,24,25,26,27,28].

### 2.5. Comparison of Outcomes

Bronchoscopic lung volume reduction demonstrated similar benefits in both PFT and symptoms when indirectly or directly compared to lung volume reduction surgery [29,30]. In terms of pulmonary function testing, primary and secondary outcomes following BLVR focused on the percentage of patient responders, defined as an increase in post-bronchodilator FEV1 percent, ranging from 10–15%, and the absolute change in post-bronchodilator FEV1 in comparison to the baseline. Reassessment in pulmonary function testing post-BLVR occurred between 3 and 12 months, depending on the study. The percentage of patient responders ranged from 37 to 59. The degree of air trapping, whether higher or lower cutoffs were used, did not result in a greater responder rate. For example, the BeLieVeR-HIFi and IMPACT trials used RV inclusion criteria of >150% predicted and ≥200% predicted; however, both studies had nearly an identical responder rate of about 39%. The majority of studies demonstrated an improvement of FEV1 by 15–20%. However, some studies, such as the EMPROVE trial, reported primarily the absolute change, which in this trial was 100 mL. The percent of responders, the percent change in FEV1, and the absolute change in FEV1 did not appear to be dependent on the spirometry or lung volume inclusion criteria. The VENT trial, however, did show that increased success was based on increasing emphysema heterogeneity and complete fissure integrity [31]. Therefore, the PFT benefits following BLVR were not dependent on the differences in inclusion criteria for FEV1, TLC, or RV, suggesting that the most inclusive range or cuffoff values taken from all of these studies can be used for candidate eligibility.

### 2.6. Comparison of Adverse Events

Pneumothorax is one of the most common and feared complications following BLVR. One of the proposed theories for the increased incidence stems from the concept of rapid expansion of the adjacent portion of the lung following the collapse of the target region. This can lead to overstretching of the expanded lung and a subsequent bronchoalveolar fistula. This has been reported to occur predominately within the first 3 days following BLVR. The incidence of pneumothorax in all the reported studies varied from 4.2–34.4% (Table 2) [32,33]. Death rates as a complication of the procedure are reportedly low in all studies, ranging from 0–3% [33]. The exact mechanism for the increased risk of early pneumothorax following BLVR has not been entirely elucidated; however, a recent study evaluating the incidence of pneumothorax following BLVR found that decreasing the fractional inspired oxygen (FIO2) during the procedure led to a significant reduction in pneumothorax [34]. In all the aforementioned studies, the PFT inclusion criteria did not appear to impact the incidence of pneumothorax or mortality. For example, the LIBERATE, TRANSFORM, and IMPACT trials used nearly identical cutoff values for the post-bronchodilator FEV1 and total lung capacity for inclusion criteria; however, they differed in the residual volume cutoff values (≥175% predicted, ≥180% predicted, and ≥200% predicted, respectively). Despite the differences in RV criteria, the incidence of pneumothorax for LIBERATE, TRANSFORM, and IMPACT was 34.4%, 29.2%, and 25.6%, respectively. At first, this suggests that higher RV cutoffs are associated with lower pneumothorax incidence; however, the VENT, REACH, and BeLieVeR-HIFi studies boast the lowest pneumothorax rates at 4.2%, 7.6%, and 8%, respectively. In addition, the STELVIO and IMPACT trials both used the same TLC cutoff values and homogenous emphysema (but differed in post-bronchodilator FEV1 inclusion criteria); however, despite a higher RV cutoff for the IMPACT trial (≥200% predicted vs. >150% predicted), the incidence of pneumothorax was greater in the IMPACT trial than the STELVIO trial (25.6% vs. 18%) [15,16,21,22,23,24,25,26,27,28]. Since higher or lower inclusion cutoff values for post-bronchodilator FEV1, TLC, and RV did not result in an increased incidence of mortality or pneumothorax, these comparisons between studies suggest that a broader inclusion criterion is feasible without imparting an increased risk of adverse events. Liberalizing the PFT inclusion criteria would result in a higher number of referrals and qualifying patients who may benefit from the procedure.

## 3. Discussion

### Proposed Spirometry and Lung Volume Inclusion Criteria

Optimization of patient care calls for expanding access to evidence-based therapies that improve patient-centered outcomes while minimizing detrimental adverse effects. Bronchoscopic lung volume reduction has been incorporated into the GOLD guidelines for the management of severe COPD, which remains symptomatic despite optimal medical therapies. However, the recommendation for BLVR comes without detailed criteria for patient selection. In this review of the existing randomized controlled trials for bronchoscopic lung volume reduction, it appears that including patients with FEV1 between 45 and 60%, TLC ≥ 100%, and RV of 150 to 200% of predicted values does not have a significant impact on outcomes or adverse events while also increasing accessibility to the procedure when maximal medical therapy has failed to adequately manage symptoms of severe emphysema.

Determining who will benefit from BLVR and how to best quantify that benefit remains a topic of contention. In consideration of the hallmark studies on BLVR, improvement in spirometry and functional assessments were included. Considering the hallmark studies in BLVR, the variability of the inclusion criterion between the prospective trials did not appear to impact the outcome in terms of the change in post-bronchodilator FEV1. The incidence of pneumothorax or adverse events was also not significantly different when compared with the variations between studies. Based on these findings, we propose that the inclusion criteria for BLVR based on pulmonary function testing, specifically, can be based on the most inclusive criteria according to the studies analyzed. Nearly all studies utilized a TLC > 100% predicted, and therefore, this was maintained as the preferred cutoff. We suggested increasing the post-bronchodilator FEV1 cutoff to <60% based on the STELVIO and BeLieVeR-HIFi trials, which had comparable response rates and pneumothorax incidence compared to all other trials. We opted for an RV inclusion criterion of >150% predicted since this was used in the majority of the studies, allows for the largest inclusion, and had comparable outcomes and pneumothorax incidence compared to the LIBERATE, IMPACT, and TRANSFORM trials [12,13,14,15,16,17,18,19,20,21,22,23,24,25,26,27,28].

Therefore, our proposed BLVR inclusion criterion for spirometry and lung volume parameters includes an FEV1 of 15–60% predicted, TLC ≥ 100% predicted, RV ≥ 150% predicted, and DLCO ≥ 20% predicted. These thresholds are intended to standardize and streamline the referral and evaluation process while maximizing access to a therapy shown to improve symptoms, physiology, and longevity. These proposed spirometric, lung volume, and diffusion criteria should not replace comprehensive patient evaluations. We reiterate that clinicians should continue to use fissure integrity, heterogeneity of the emphysema, assessment of collateral ventilation, presence of lung nodules, and pulmonary hypertension, among other clinical parameters, when determining eligibility for BLVR, as these parameters have been shown to be predictors of success or complications.

## 4. Conclusions

Bronchoscopic lung volume reduction fills a much-needed gap in healthcare for patients with severe COPD who remain symptomatic on maximal medical therapy. These patients may or may not be candidates for surgical lung volume reduction or lung transplant. In either case, BLVR provides a minimally invasive treatment option prior to or in place of surgical intervention.

Bronchoscopic lung volume reduction has demonstrated a reduction in morbidity and mortality in patients with severe emphysema. Studies have differed regarding the cutoff parameters used for the inclusion criterion. While there are other inclusion and exclusion criteria, PFT values are often utilized as screening tests for candidacy for this procedure. The use of broader PFT inclusion criteria for eligibility of bronchoscopic lung volume reduction does not appear to impact the responder rate or adverse effects. Expanding the inclusion criteria based on the post-bronchodilator FEV1, TLC, and RV percent predicted may result in increased patient referrals while maximizing eligibility for BLVR.

## Figures and Tables

**Table 1:** Prospective study inclusion criteria. FEV1 = post-bronchodilator forced expiratory volume at 1 s; TLC = total lung capacity; RV = residual volume; DLCO = diffusion capacity of carbon monoxide. All percentages are predicted according to the criteria used in the respective study.

Study	FEV1	TLC	RV	DLCO
VENT	15–45%	>100%	>150%	≥20%
STELVIO	<60%	>100%	>150%	No mention
BeLieVer-HIFi	<50%	>100%	>150%	No mention
IMPACT	≥15%, <45%	>100%	≥200%	No mention
TRANSFORM	15–45%	>100%	≥180%	No mention
LIBERATE	15–45%	>100%	≥175%	≥20%
EMPROVE	<45%	≥100%	≥150%	No Mention
REACH	<45%	≥100%	≥150%	No Mention

**Table 2:** Complication rates. Rates were reported as the longest follow-up available at the time of the index study (3, 6, or 12 months).

Study	Mortality	Pneumothorax
VENT	3.7%	4.2%
STELVIO	3%	18%
BeLieVer-HIFi	8%	8%
IMPACT	0%	25.6%
TRANSFORM	1.5%	29.2%
LIBERATE	3.9%	34.4%
EMPROVE	5.3%	28.3%
REACH	0%	7.6%
